# Ultra-Processed Food Consumption and Metabolic-Dysfunction-Associated Steatotic Liver Disease (MASLD): A Longitudinal and Sustainable Analysis

**DOI:** 10.3390/nu17030472

**Published:** 2025-01-28

**Authors:** Silvia García, Margalida Monserrat-Mesquida, Lucía Ugarriza, Miguel Casares, Cristina Gómez, David Mateos, Escarlata Angullo-Martínez, Josep A. Tur, Cristina Bouzas

**Affiliations:** 1 Research Group on Community Nutrition and Oxidative Stress, University of the Balearic Islands-IUNICS, 07122 Palma de Mallorca, Spain; silvia.garcia@uib.es (S.G.); margalida.monserrat@uib.es (M.M.-M.); cristina.gomez@ssib.es (C.G.); cristina.bouzas@uib.es (C.B.); 2 Health Research Institute of Balearic Islands (IdISBa), 07120 Palma de Mallorca, Spain; 3 CIBEROBN (Physiopathology of Obesity and Nutrition CB12/03/30038), Institute of Health Carlos III, 28029 Madrid, Spain; 4 Primary Health Care Center Camp Redó, IBSalut, 07010 Palma de Mallorca, Spain; 5 Radiodiagnosis Service, Red Asistencial Juaneda, 07011 Palma de Mallorca, Spain; 6 Clinical Analysis Service, University Hospital Son Espases, 07120 Palma de Mallorca, Spain; 7 Hospital of Manacor, 07500 Manacor, Spain; 8 Primary Health Care Center Escola Graduada, IBSalut, 07001 Palma de Mallorca, Spain

**Keywords:** metabolic-dysfunction-associated steatotic liver disease, ultra-processed foods, sustainability, liver fat content, dietary patterns, Mediterranean diet

## Abstract

Background: The rising prevalence of metabolic-dysfunction-associated steatotic liver disease (MASLD) is a significant health challenge, and the consumption of ultra-processed foods (UPFs) could play a key role. Aim: The aim is assess the impact of UPF consumption changes on the development and progression of MASLD in adults. Design: This is a longitudinal study to assess how changes in UPF consumption affect liver fat and MASLD parameters over 6 months in 70 participants. Methods: Dietary intake was assessed using a validated food frequency questionnaire, and foods were classified according to the NOVA system. Participants were divided into three groups based on UPF consumption changes: maximum (T1), medium (T2), and minimum reduction (T3). Fatty liver parameters were assessed with magnetic resonance imaging and ultrasonography. Mediterranean diet (Med-diet) adherence and sociodemographic parameters were also recorded. The General Linear Model was used to determine relationships between UPF consumption, fatty liver disease parameters, and diet. Results: Participants in T1 experienced a 7.7% reduction in intrahepatic fat content (IFC) compared to 2.6% in T3. T1 showed increased Med-diet adherence and decreased meat and sweets consumption. The energy intake decreased by 605.3 kcal/day in T1, while T3 showed an increase of 209.5 kcal/day. Conclusions: Reducing UPF consumption leads to a decrease in IFC, associated with high Med-diet adherence and low calorie intake. Adopting these dietary patterns aligns with global sustainability goals and could further benefit MASLD patients by addressing environmental challenges alongside improving liver health.

## 1. Introduction

The rising prevalence of metabolic-dysfunction-associated steatotic liver disease (MASLD) has prompted significant concern among healthcare professionals and researchers [1]. A growing body of evidence suggests that one of the key contributors to this condition is the widespread consumption of ultra-processed foods (UPFs), which are linked to MASLD through a variety of metabolic and epidemiological mechanisms [2].

MASLD, previously known as non-alcoholic fatty liver disease (NAFLD) [3], is characterized by the accumulation of fat in the liver in individuals who do not consume excessive amounts of alcohol [4]. This condition can progress to steatohepatitis, fibrosis, and eventually cirrhosis of the liver [3,5]. According to data from the Institute for Health Metrics and Evaluation, NAFLD, currently MASLD, resulted in approximately 3.67 million global disability-adjusted life years (DALYs) in 2021, with an increase in DALYs within the age range of 30 to 85 years, for both sexes.

High-income countries exhibit the highest DALYs. DALYs are a measure of overall disease burden, expressed as the number of years lost due to ill health, disability, or early death. Specifically, cirrhosis and liver cancer caused by MASLD were responsible for 72.5% and 27.5% of the DALYs, respectively [6]. As shown by data from 2018 to 2021, the trend in MASLD-related deaths has been increasing, with the 2021 data showing 0.138 million deaths globally, affecting both women and men similarly [6,7]. These figures emphasize the substantial global health burden of MASLD, highlighting the need to address preventable risk factors like the consumption of UPFs.

UPFs are industrially manufactured food products that are high in added sugars, saturated fats, salt, and additives while being low in essential nutrients and fiber [8]. Excessive UPF consumption contributes to obesity, insulin resistance, and hepatic inflammation, key risk factors for MASLD [8,9].

Due to their high caloric density and low nutrient content, UPFs contribute to increased total caloric intake, leading to weight gain and obesity [9]. Obesity is a well-established risk factor for fat accumulation in the liver and the development of insulin resistance, which are critical initial steps in the pathogenesis of MASLD [3].

Research has shown that the high consumption of UPFs among children and adolescents is linked to an increased incidence of MASLD and other metabolic conditions [10]. Similarly, it has been observed that in both adult and elderly populations, a diet high in UPFs increases the risk of developing fatty liver and its long-term complications [11,12].

The biological mechanisms linking UPF consumption with MASLD involve several processes. First, UPFs often contain high levels of fructose, which is metabolized in the liver and can promote hepatic lipogenesis, thereby increasing fat accumulation in the liver [13]. The negative effects of fructose in UPFs are amplified by other components, such as saturated fats, food additives, and the absence of fiber and essential nutrients, which stimulate de novo lipogenesis and exacerbate metabolic dysfunction [14]. Second, UPFs can induce oxidative stress and inflammation, which are key factors in the progression of MASLD to more advanced states like steatohepatitis [15]. Third, the poor nutritional quality of UPFs, being deficient in fiber and antioxidants and high in additives, can disrupt the balance of the gut microbiome, leading to harmful health effects [16].

Currently, there are no medical treatments that directly cure MASLD, making lifestyle factors, particularly diet, essential for its management [17,18].

This study underscores the importance of reducing UPF consumption as a preventive dietary strategy to slow MASLD progression. Specifically, we aim to explore the relationship between UPF intake and metabolic indicators, such as liver fat accumulation and weight gain, in adult populations.

## 2. Methods

### 2.1. Design

**Figure 1:**
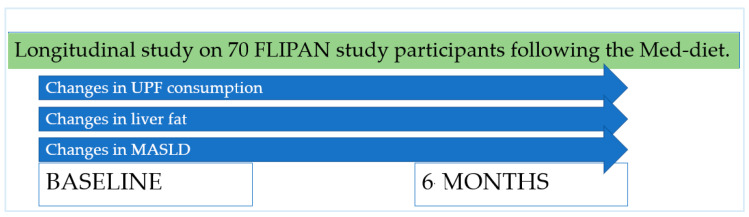
Design of the present study.

The present study is a longitudinal analysis within the frame of the FLIPAN study, registered at ClinicalTrials.gov (https://clinicaltrials.gov/ct2/show/NCT04442620; accessed 22 February 2022) with the identifier NCT04442620 [19]. The FLIPAN study is a prospective randomized trial conducted across multiple centers in Spain, featuring personalized nutritional counseling based on a customized Mediterranean diet (Med-diet), combined with physical activity promotion, aimed at preventing and reversing metabolic-dysfunction-associated fatty liver disease (MASLD) among patients with metabolic syndrome (MetS). A schema explaining the design of the present study can be seen in Figure 1.

### 2.2. Participants, Recruitment, and Ethics

The recruitment of the FLIPAN study participants was conducted between June 2018 and January 2020. In total, 143 people were contacted. Participants were required to be between 40 and 60 years old, be characterized as overweight or obese (with a body mass index of between 27 and 40 kg/m^2^), be diagnosed with MASLD using magnetic resonance imaging (MRI), and meet at least three MetS criteria according to the International Diabetes Federation guidelines [20] to be included.

Participants were excluded if they had a history of cardiovascular disease, liver disease (excluding MASLD), cancer or malignancy within the past five years, haemochromatosis, prior bariatric surgery, untreated depression, alcohol or drug abuse, pregnancy, primary endocrinological disorders (except untreated hypothyroidism), severe psychiatric disorders (including schizophrenia, bipolar disorder, eating disorders, or depression requiring hospitalization within the past six months), a Beck Depression Inventory score above 30, or concurrent steroid therapy or if they were unable to provide informed consent.

**Figure 2:**
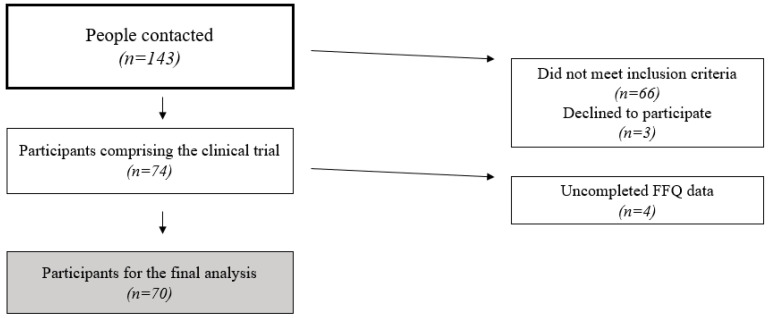
Participant eligibility process flow-chart.

After excluding participants who did not meet the criteria or chose not to participate, 74 participants took part in the clinical trial. Four additional participants were excluded because they did not complete the food frequency questionnaire (FFQ) in one of the two visits. This questionnaire is used in the present study to assess UPF consumption. A total of 70 participants (*n* = 70) were included in the definitive analysis. A flow-chart of the eligibility process can be seen in Figure 2.

Participants were recruited in primary healthcare centers through general practitioners, who assessed whether the inclusion criteria were met (except for the MRI criteria, which were determined in a private clinic). This recruitment strategy, conducted through primary healthcare settings and standardized screening processes, strengthens the representativeness of the sample by ensuring a broad and diverse participant pool from the target population.

This study adhered to the ethical standards outlined in the Declaration of Helsinki and was approved by the Research Ethics Committee of the Balearic Islands (ref. IB 2251/14 PI; 26 February 2014). Participants were thoroughly informed about the study protocol and provided written consent.

### 2.3. Sociodemographic Characteristics

Sociodemographic data were collected via questionnaires, focusing on participants’ sex, age, educational level (primary school, secondary school, or university), and employment status (non-working, working, or retired). Information on physical activity levels was collected using the Minnesota Leisure Time Physical Activity Questionnaire, with energy expenditure expressed as the metabolic equivalents of tasks (min/week) [21,22].

### 2.4. Fatty Liver Disease Parameters

Abdominal MRI, with a 12-channel phased-array coil (Signa Explorer 1.5T, General Electric Healthcare, Chicago, IL, USA), was employed to quantify liver fat as the mean percentage, determined as intrahepatic fat content (IFC) (23). A more detailed explanation of this procedure, as well as its reliability and significance, is provided elsewhere [23,24,25].

Additionally, ultrasonography methods were used to evaluate the hepatic steatosis level. The process included a visual assessment of liver echogenicity quality, a visual comparison of the echo amplitude difference between the kidneys and the liver, and an evaluation of the clarity of the liver’s blood vessel structures. The clinical classification used a 4-point scale, less than 5% (grade 0), 5–33% (grade 1), 33–66% (grade 2), and greater than 66% (grade 3), as described elsewhere [26]. The hepatic fibrosis level and tissue stiffness (measured in kilopascals, kPa) were evaluated using transient elastography (FibroScan^®^, Echosens, París, France), with the participant lying down in a resting respiratory position, with the right arm elevated above the head for optimal intercostal access. Anthropometry was determined by bioimpedance with a segmental body composition analyzer (Tanita MC780P-MA, Tanita, Tokyo, Japan) and was utilized to measure weight (kg) and visceral fat quantity (13 being the cut-off point between low and high values).

### 2.5. Dietary Parameters

A validated 143-item semi-quantitative FFQ [27,28,29], administered by trained dietitians, assessed the participants’ usual dietary intake at baseline and after 6 months, with these visits also used to provide personalized follow-up and promote healthy lifestyle habits aligned with the Med-diet. Each food item in the FFQ had a standardized portion size, and consumption frequencies were recorded on a scale ranging from never or almost never to more than six times per day. The reported frequencies were converted into daily intake in grams by multiplying them by the portion size weight. The quantity of grams consumed per food group was calculated, including the following food groups: vegetables, fruits, legumes, cereals, dairy, meat, olive oil, fish, nuts, and sweets and pastries. The energy intake per person per day (kcal/day) was calculated using a computer program with data from Spanish food composition tables [30,31], multiplying the grams of each food item by its respective energy content in 100 g and finally adding all food items consumed. The Med-diet adherence was also reported. A previously validated 17-item energy-reduced Med-diet questionnaire was administered at baseline and after six months [32,33]. Each item of the Med-diet questionnaire corresponds to a characteristic of the Med-diet, with participants scoring 1 for adherence and 0 for non-adherence. Scores range up to 17 points, reflecting higher adherence and diet quality over time.

### 2.6. Ultra-Processed Food Consumption Assessment

The dietary information extracted from the FFQ was used to determine UPF consumption according to the NOVA system. This system was developed in 2010 by the NUPENS research group at the School of Public Health of the University of Sao Paulo, under the leadership of Dr. Carlos Monteiro. It classifies foods and beverages based on their level of processing and consists of four main groups: NOVA 1: unprocessed or minimally processed foods; NOVA 2: processed culinary ingredients; NOVA 3: processed foods; and NOVA 4: UPFs [8,34]. Each food item included in the FFQ was categorized according to these four NOVA groups. Following previous methodologies [35,36], the percentage of UPF consumption was calculated at both baseline and after 6 months. This was achieved by dividing the total grams of UPF consumed by the total grams of all food items consumed daily and multiplying by 100.

### 2.7. Statistics

Analyses were conducted using SPSS statistical software version 27.0 (SPSS Inc., Chicago, IL, USA). Data are presented as the mean and standard deviation (SD), except for prevalence data, which are reported as the sample size and percentage. The Chi-squared test was employed for categorical variables, while one-way ANOVA was applied to continuous variables. To create our tertiles, we first calculated the UPF consumption and its percentage at each time point, as described in the previous section, and then assessed the changes in these percentages from baseline to 6 months. These changes were categorized into tertiles to reflect different levels of reduction: Tertile 1 (T1) represents the group with the highest reduction in UPF consumption, defined as a decrease of 7.27% or more (≤−7.27). Tertile 2 (T2) includes participants with a moderate reduction, with values ranging from −7.26% to −0.63% (from −7.26 to −0.63). Finally, Tertile 3 (T3) represents the smallest reduction in UPF consumption, with changes of 0.62% or less (≥−0.62). This approach allows us to differentiate participants based on the extent of their reduction in UPF consumption. The General Linear Model (GLM) was used to assess the relationships between changes in UPF consumption percentages, fatty liver disease parameters, and diet parameters over the 6-month period, with adjustments for the Homeostatic Model Assessment of Insulin Resistance (HOMA-IR) and physical activity represented by metabolic equivalent tasks. Given that the study participants had MetS, including insulin resistance, obesity, and glucose intolerance, adjusting for the HOMA-IR helps to isolate the effects of UPF consumption on MASLD, reducing biases related to glucose levels and obesity. Bonferroni’s post hoc test was applied to identify statistically significant differences (*p* < 0.05) between groups across time (a, b, c), across time within groups (*), and between the time* group interactions.

## 3. Results

A total of 70 participants were included in the final analysis. These participants were separated into three groups according to UPF consumption. Table 1 shows the three groups mentioned: T1: participants with a maximum reduction in UPF% consumption (*n* = 23); T2: participants with a medium reduction in %UPF consumption (*n* = 24); and T3: participants with a minimum reduction in %UPF consumption (*n* = 23).

Table 1 also shows the descriptive characteristics of the sample according to changes in UPF% consumption within a period of 6 months. The studied sociodemographic variables were sex, educational level, job situation, age, and the metabolic syndrome parameters. A higher number of men were seen in all of the three tertiles, the educational level was similar between groups, and most participants were of an employed status, with the youngest participants showing the greatest reduction in percentage UPF consumption over the 6-month period with a mean (and standard deviation) of 50.8 (6.9) years old. None of the descriptive variables showed significant differences between groups (*p* < 0.05).

Table 2 shows the fatty liver parameters according to 6-month changes in UPF percentage consumption.

Weight and visceral fat were also assessed and are shown in Table 2. IFC showed statistical significance when related to changes in UPF% consumption over 6 months, with a reduction of 7.7% in IFC in the group with the maximum reduction in %UPF consumption (T1) compared to a 2.6% reduction in the group with the minimum reduction (T3). The size effect of IFC was η^2^*p* = 0.33. The other fatty liver parameters showed minor changes between groups but were not statistically significant.

Table 3 shows the Med-diet adherence, the Med-diet food groups, and NOVA food groups according to 6-month changes in UPF consumption.

Med-Diet adherence was associated with a reduction in UPF% consumption. Hence, higher adherence to the Med-diet aligns with a more substantial reduction in UPF%. T1 increased its Med-diet adherence by a mean of 5.2 points. Significant changes were observed in the Med-diet food groups for meat, nuts, and sweets and pastries. A notable reduction in meat (−68.8 g/day) and sweets and pastries consumption (−23.7 g/day) was seen in T1 compared to T3. Nut consumption increased in all three groups. All NOVA food groups showed significant changes, except for non-processed foods. The greatest reduction was in UPFs, with a reduction of −399.8 g/day in T1 compared to an increase of 41.1 g/day in T3. All three groups reduced their high-processed food consumption, with a small reduction in low-processed foods in T1 and T2 (−19.8 g/day and −4.4 g/day, respectively) compared to an increase of 5.2 g/day in T3. The total energy intake was significantly related to the reduction in UPF% consumption, with a reduction of 605.3 kcal/day in T1, compared to a reduction of 89.2 kcal/day in T2 and an increase of 209.5 kcal/day in T3. The size effects calculated with η^2^*p* were 0.85, 0.63, 0.32, 0.27, 0.62, 0.54, 0.46, and 0.86 for Med-diet adherence, meat, nuts, sweets and pastries, low-processed foods, high-processed foods, UPFs, and total energy intake, respectively.

## 4. Discussion

A reduction in UPF consumption is a beneficial factor in improving MASLD parameters, including a significant decrease in IFC. Dietary changes, including a reduction in meat, sweets, and pastries and an overall improvement in Med-diet adherence, have been identified as factors contributing to reductions in UPF consumption and IFC. These results emphasize the importance of adopting dietary patterns that focus on minimally processed foods. Beyond health benefits, shifting to minimally processed foods has substantial implications for environmental sustainability. UPF production involves the extensive use of natural resources and often generates considerable amounts of waste, contributing significantly to global environmental degradation [37]. This is important to mention because a reduced consumption of UPF could help both planetary health and population health at the same time.

A remarkable reduction in total energy intake was seen when reducing UPF consumption, potentially being one of the factors contributing to the IFC reduction. This finding is consistent with previous research [38,39,40]. Specifically, one of the studies highlighted that energy intake is a key factor in decreasing fat accumulation in the liver [40]. An increase in calorie intake can be harmful, resulting in an imbalance between energy intake and energy expenditure, meaning that the calories consumed exceed the individual’s needs, leading primarily to overweight and obesity, among other health risks frequently linked to MASLD [17,41]. These results reinforce the idea that reducing UPF consumption can directly lower energy intake, facilitating weight loss and improvements in liver parameters.

A recent report from the Global Food Research Program, called “A global threat to public health”, determined that UPF consumption currently accounts for over half of the estimated total caloric intake in the United States, the United Kingdom, and Canada and approximately 20–40% of the caloric intake in other high- and middle-income countries, with sales increasing rapidly each year [42]. Decreasing the UPF intake can directly lead to a decrease in energy consumption and, consequently, a reduction in body weight and obesity [43].

UPFs are typically energy-dense and nutrient-poor, promoting caloric excess while disrupting metabolic pathways and increasing systemic inflammation and oxidative stress, all of which exacerbate MASLD [35,44]. UPFs can influence the homeostatic mechanisms of body weight regulation and create an intestinal environment conducive to the proliferation of microorganisms that promote inflammatory diseases [45,46]. The artificial additives and preservatives present in UPFs can disrupt the gut microbiome, leading to dysbiosis and the disruption of the intestinal mucus barrier [16,47]. Additionally, UPFs are typically low in dietary fiber, which is an essential nutrient for the growth and activity of beneficial gut microorganisms. By reducing the intake of UPFs, the consumption of fiber-rich foods such as fruits, vegetables, and whole grains can be increased, thereby providing nourishment for beneficial bacteria, which in turn can support liver health by reducing intestinal permeability, preventing the systemic inflammatory response and restoring microbial balance [48].

A reduction in UPF consumption has been associated with improvements in liver health, potentially through other mechanisms. First, reducing UPF intake can lower systemic inflammation, a key factor contributing to MASLD. UPFs, due to their high content of synthetic and pro-inflammatory compounds, can exacerbate liver inflammation, and their reduction may alleviate this [15,44,49]. Second, dietary patterns that focus on minimally processed foods and are rich in anti-inflammatory and antioxidant compounds, such as polyphenols and omega-3 fatty acids, can help improve liver fat metabolism and reduce hepatic fat accumulation [50]. Among them, the Med-diet has proven to be especially beneficial, being recognized as useful for the prevention and management of MASLD [51,52]. In the current study, the Med-diet appears to be related to a reduction in UPF consumption, reflecting how following the Med-diet could be a strategy to decrease UPFs and therefore reduce IFC in patients with MASLD. The Med-diet, rich in whole, minimally processed foods such as fruits, vegetables, whole grains, legumes, nuts, and fish, naturally limits the intake of UPFs and supports liver health through its anti-inflammatory and antioxidant properties [53,54].

By improving diet quality, the Med-diet helps reduce liver fat, making it a beneficial dietary approach for individuals with MASLD. Within the products of the Med-diet, the reduced consumption of meat products, sweets, and pastries was associated with a higher reduction in UPF consumption. This reduction in red and processed meats, sweets, and pre-cooked products was also observed in a previous study, which demonstrated that changes in UPF consumption were linked to increased adherence to the Med-diet, as well as reductions in weight, BMI, and energy intake. Additionally, the study highlighted how these dietary changes led to decreases in environmental strain, such as CO_2_ emissions and energy use [36].

UPFs contribute significantly to environmental damage, mainly due to the greenhouse gas emissions from their production [36,55]. As their consumption increases, it creates a dually negative impact on both consumer and environmental health [56]. Adopting a Med-diet pattern, which emphasizes local, seasonal, and plant-based foods that generally require fewer resources than UPFs, red meat products, and sweets, could provide dual benefits for the environment and public health [57]. Research has demonstrated that lower-CO_2_-emission diets, which emphasize minimally processed foods and the reduced consumption of UPFs, contribute to improved outcomes for MetS and glycemic control [58]. The shift to these dietary patterns would also benefit MASLD patients, given the shared pathophysiological mechanisms [59].

When proposing a diet for MASLD patients, the dietary components must address associated comorbidities such as cardiovascular diseases and type 2 diabetes mellitus, as well as contributing risk factors, since these are highly interconnected [2,60]. Therefore, the current analysis has been adjusted for the HOMA-IR, to avoid bias in the results, since patients with MASLD tend to have impaired glucose levels and insulin resistance. MASLD is closely associated with insulin resistance, which is a key metabolic dysfunction linked to the development and progression of fatty liver disease. By adjusting for the HOMA-IR, we account for the impact of insulin resistance on liver health, allowing for a more accurate assessment of how UPF consumption directly affects MASLD outcomes.

This is important because individuals with higher levels of insulin resistance are more likely to develop MASLD, and insulin resistance itself can influence the way the body processes fats and sugars, contributing to liver fat accumulation [61]. Including the HOMA-IR in the analysis ensures that the relationship between UPF consumption and MASLD parameters is not confounded by the metabolic status of the participants. It helps isolate the specific impact of UPF consumption on liver health, independent of insulin resistance, providing a clearer understanding of the dietary factors contributing to MASLD. This adjustment strengthens the validity and interpretability of the findings.

Following a Med-diet pattern requires a reduction in the intake of red and processed meats as well as processed sweets and sugary beverages. These are food groups that are normally classified as ultra-processed, and their minimization is a key factor in reducing MASLD [60,62,63]. Participants who had a higher reduction in UPFs showed significant improvements across several food categories, particularly in the reduction in meat and sweets consumption, alongside increased nut intake. This result further demonstrates the important benefits of the increased consumption of minimally processed products such as fruits, vegetables, whole grains, legumes, nuts, fish, and white meats [63]. These foods contribute to achieving a healthy body weight and reducing inflammation and oxidative stress, which are crucial factors for managing MASLD [64,65,66,67].

The existing literature predominantly consists of observational studies, highlighting the need for more randomized intervention studies and longitudinal research [2]. Our longitudinal study shows how reducing UPF consumption in the diet implied a notable decrease in IFC. Similar to our findings, previously published research reported that adults with a high UPF intake exhibited a significantly elevated fatty liver index [68]. A prospective analysis, included within an RCT among Spanish adults with MASLD, demonstrated that in older adults with chronic health conditions, the consumption of UPF was directly and strongly associated with fatty liver and hepatic steatosis scores [35]. Additionally, a UK cohort study discovered that high UPF intake was linked to an increased risk of NAFLD, liver fibrosis, cirrhosis, severe liver disease, and adverse levels of multiple clinical biomarkers, showing the importance of reducing UPF intake to improve liver health [69]. This study did not, however, examine other parameters such as IFC changes over time, such as in this current study. Another study found that high UPF consumption was associated with increased odds of NAFLD in both adolescents and adults, mainly due to increased body fat. If these results are validated, lowering UPF intake could potentially prevent NAFLD, currently MASLD, in both age groups [70].

Lifestyle interventions, especially dietary changes, play a critical role in managing MASLD. These findings highlight the critical need to prioritize dietary interventions targeting UPF consumption as an effective strategy for improving liver health and preventing related conditions, simultaneously mitigating environmental impacts such as global warming and resource depletion.

### Strengths and Limitations

The current study’s strengths lie in its longitudinal design, which offers valuable insights into how changes in UPF consumption affect MASLD over a 6-month period. By employing both abdominal MRI and ultrasonography, this study ensures a comprehensive and accurate assessment of liver fat. The use of a validated 143-item semi-quantitative FFQ and the validated 17-item energy-reduced Med-diet questionnaire enhanced the precision of dietary evaluations. Moreover, the use of the NOVA system for classifying UPF consumption is a good choice because it is the most reliable and widely used system in the scientific literature. This choice allows for the effective comparison of results with other studies and ensures the accuracy and relevance of the dietary assessments. Adjusting for factors like the HOMA-IR in the analyses helps to isolate the specific impact of UPF consumption on liver health, mitigating potential biases related to glucose levels and obesity.

This study also has some limitations. The sample size of 70 participants may restrict the generalizability of the findings, as the results may not extend beyond the specific cohort studied or apply to other populations. The age of the participants (40–60 years old) could limit the extrapolation of the results to younger or older populations. The reliance on self-reported dietary intake data introduces the possibility of inaccuracies, potentially affecting the reliability of UPF consumption assessments. While the FFQ offers a general overview of dietary intake, it does not accurately capture the specific details of UPF consumption. There is a need for a validated questionnaire specifically designed to measure UPF consumption.

## 5. Conclusions

Reducing UPF consumption is beneficial for managing MASLD, as evidenced by the notable decrease in IFC among participants. This dietary shift, combined with increased adherence to the Med-diet and reduced calorie intake, can provide a dietary path to reducing adverse liver health outcomes. Moreover, adopting these dietary patterns aligns with global sustainability goals and could further benefit MASLD patients by addressing environmental challenges alongside improving liver health. Given the significant role of diet in MASLD management, the current lack of medical treatments, and the impact of UPF consumption on environmental health, our findings underscore the need for dietary interventions that specifically target UPF reduction, therefore improving liver health, mitigating the associated risks, and simultaneously enhancing planet sustainability.

## Figures and Tables

**Table 1:** Descriptive characteristics of the sample according to changes in UPF consumption (%) in 6 months.

	Maximum Reduction in %UPF Consumption ^§^ *n* = 23	Medium Reduction in %UPF Consumption ^§^ *n* = 24	Minimum Reduction in %UPF Consumption ^§^ *n* = 23	*p* Value
Sex Men Women		*n* (%)		
	15 (65.2)	16 (66.7)	13 (56.5)	0.741
	8 (34.8)	8 (33.3)	10 (43.5)	
Educational Level Primary Secondary University		*n* (%)		
	8 (34.8)	9 (37.5)	9 (39.2)	0.798
	9 (39.1)	11 (45.8)	7 (30.4)	
	6 (36.1)	4 (16.7)	7 (30.4)	
Job situation Not working Working Retired		*n* (%)		
	3 (13.0)	4 (16.7)	4 (17.4)	0.651
	17 (73.9)	17 (70.8)	18 (78.3)	
	3 (13.0)	3 (12.5)	1 (4.3)	
		Mean (SD) [CI]		
Age (years)	50.8 (6.9) [47.8, 53.7]	54.8 (6.7) [51.9, 57.6]	52.4 (6.5) [49.5, 55.2]	0.132
Glucose (mL/dL)	108.4 (17.9)	112.1 (19.8)	127.2 (72.9)	0.320
HbA1c (%)	5.9 (0.8)	6.1 (0.8)	6.3 (2.1)	0.663
HDL (mg/dL)	40.5 (7.3)	41.1 (8.9)	42.7 (11.1)	0.696
TG (mg/dL)	210.7 (78.9)	255.3 (441.5)	199.1 (137.8)	0.760
WC (cm)	113.6 (8.1)	113.7 (9.6)	110.9 (9.4)	0.485
BPsyst (mmHg)	135.4 (15.4)	138.4 (15.8)	137.4 (19.9)	0.832
BPdias (mmHg)	81.1 (10.2)	84.3 (8.1)	81.2 (8.1)	0.380

Abbreviations: UPF: ultra-processed food. SD: standard deviation. CI: confidence interval. HbA1c: glycosylated hemoglobin. HDL: high-density lipoprotein cholesterol. TG: triglyceride. WC: waist circumference. BPsyst: systolic blood pressure. BPdias: diastolic blood pressure. ^§^ Differences in %UPF consumption between baseline and at 6 months follow-up distributed across tertiles. Confidence intervals are calculated at the 95% confidence level and are represented by the values in brackets []. Differences in means between groups were tested by one-way ANOVA. Differences in prevalences across groups were examined using χ^2^.

**Table 2:** Fatty liver parameters according to 6-month changes in percentage (%) of UPF consumption.

		Maximum Reduction in %UPF Consumption ^§^ *n* = 23	Medium Reduction in %UPF Consumption ^§^ *n* = 24	Minimum Reduction in %UPF Consumption ^§^ *n* = 23	Time * Group
		Mean (SD) [CI]	Mean (SD) [CI]	Mean (SD) [CI]	
Intrahepatic fat content (%)	Baseline 6 months ▲	17.9 (10.5) [13.4, 22.5] 10.3 (6.1) [7.6, 12.9] −7.7 (8.4) * ^d^ [−11.3, −4.1]	15.1 (9.8) [10.9, 19.3] 11.8 (8.4) [7.7, 15.8] −3.1 (5.8) ^d^ [−5.9, −0.1]	15.1 (9.7) [10.8, 19.2] 12.4 (7.5) [8.7, 16.1] −2.6 (8.9) [−6.9, 1.6]	0.047 ^
Steatosis level (grades)	Baseline 6 months ▲	1.9 (0.3) [1.7, 2.1] 1.3 (0.8) [0.9, 1.7] −0.6 (0.9) * [−1.1, −0.1]	1.8 (0.5) [1.5, 2.1] 1.6 (0.7) [1.2, 1.9] −0.2 (0.8) [−0.6, 0.2]	1.7 (0.5) [1.5, 1.9] 1.6 (0.8) [1.1, 2.1] −0.3 (0.6) [−0.6, 0.1]	0.546
Fibrosis level (grades)	Baseline 6 months ▲	1.1 (0.4) [0.8–1.2] 1.1 (0.5) [0.8–1.3] 0.1 (0.4) [−0.1, 0.2]	1.3 (0.9) [0.8, 1.7] 1.2 (0.8) [0.7, 1.5] −0.1 (0.6) [−0.5, 0.2]	1.3 (1.1) [0.8, 1.8] 1.7 (1.3) [0.8, 2.5] 0.8 (1.5) [−0.2, 1.7]	0.197
Stiffness of liver tissue (kPa)	Baseline 6 months ▲	4.7 (1.1) [4.3, 5.2] 5.1 (1.3) [4.4, 5.7] 0.2 (1.3) [−0.4, 0.8]	5.3 (1.8) [4.4, 6.1] 5.4 (1.5) [4.6, 6.1] −0.1 (1.2) [−0.7, 0.7]	5.1 (1.5) [4.4, 5.8] 5.7 (2.1) [4.4, 6.9] 0.8 (2.4) [−0.7, 2.3]	0.849
Weight (kg)	Baseline 6 months ▲	92.5 (11.8) [87.3, 97.4] 87.6 (11.1) [82.8, 92.4] −4.8 (3.5) * [−6.3, −3.3]	95.2 (16.1) [88.4, 102, 1] 91.9 (15.9) [85.1, 98.5] −3.4 (4.5) * [−5.2, −1.4]	94.5 (13.3) [88.7, 100.1] 91.9 (13.1) [86.3, 97.6] −2.5 (4.3) * [−4.3, −0.5]	0.082
Visceral fat (points)	Baseline 6 months ▲	13.5 (3.4) [12.1, 15.1] 12.8 (3.1) [11.4, 14.1] −1.1 (1.1) * [−1.6, −0.6]	15.1 (3.7) [13.4, 16.7] 14.3 (3.5) [12.7, 15.7] −0.6 (1.4) * [−1.2, −0.1]	13.1 (3.5) [11.5, 14.5] 12.6 (3.2) [11.2, 13.9] −0.4 (1.3) [−1.1, 0.1]	0.101
BMI (kg/m^2^)	Baseline 6 months ▲	33.1 (3.1) [31.7, 34.4] 31.7 (2.7) [30.4, 33.2] −1.4 (1.1) * [−2.1, −0.8]	34.5 (4.1) [32.7, 36.3] 32.3 (3.6) [30.6, 34.1] −1.1 (1.7) * [−2.1, −0.3]	33.4 (4.3) [31.5, 35.4] 31.8 (4.1) [30.1, 33.7] −1.1 (1.6) * [−1.8, −0.3]	0.697

Abbreviations: UPF: ultra-processed food. SD: standard deviation. CI: confidence interval. BMI: body mass index (kg/m^2^). ^§^ Differences in %UPF consumption between baseline and at 6 months follow-up distributed across tertiles. Confidence intervals are calculated at the 95% confidence level and are represented by the values in brackets []. ▲ Change between baseline and 6 months. ^ Size effect was calculated using η^2^*p* (partial eta squared). * Time-related changes within the group. (d): letters denote intergroup differences in time-related changes (post hoc analysis). GLM was adjusted by HOMA-IR and physical activity.

**Table 3:** Diet parameters and NOVA food groups according to 6-month changes in percentage (%) of UPF consumption.

		Maximum Reduction in %UPF Consumption ^§^ *n* = 23	Medium Reduction in %UPF Consumption ^§^ *n* = 24	Minimum Reduction in %UPF Consumption ^§^ *n* = 23	Time * Group
		Mean (SD) [CI]	Mean (SD) [CI]	Mean (SD) [CI]	
Adherence to Med-diet (points)	Baseline 6 months ▲	7.4 (2.7) [6.1, 8.5] 12.6 (2.7) [11.4, 13.7] 5.2 (2.9) * ^e^ [3.9, 6.4]	8.4 (2.7) [7.2, 9.5] 12.3 (2.7) [11.1, 13.3] 3.8 (2.9) * [2.6, 5.1]	8.7 (2.3) [7.6, 9.7] 11.2 (2.4) [10.1, 12.2] 2.5 (2.8) * ^e^ [1.3, 3.7]	0.013 ^
Vegetables (g/day)	Baseline 6 months ▲	242.7 (117.5) ^b^ [191.1, 293.4] 322.8 (128.9) [267.1, 378.5] 80.2 (116.5) * ^e^ [29.7, 130.5]	298.9 (163.1) [230.1, 367.7] 371.7 (147.2) [309.5, 433.8] 72.8 (171.4) * [0.41, 145.1]	408.4 (215.9) ^b^ [315.1, 501.8] 395.6 (214.7) [302.7, 488.4] −12.7 (109.5) ^e^ [−60.1, 34.5]	0.055
Fruits (g/day)	Baseline 6 months ▲	258.4 (184.5) [178.6, 338.2] 279.2 (164.7) [207.9, 350.3] 20.7 (175.1) [−54.9, 96.4]	311.7 (232.8) [213.4, 410.1] 326.2 (195.7) [243.5, 408.8] 14.5 (154.8) [−50.9, 79.8]	347.3 (211.6) [255.8, 438.8] 399.6 (256.1) [288.9, 510.3] 52.3 (180.2) [−25.6, 130.2]	0.875
Legumes (g/day)	Baseline 6 months ▲	23.5 (14.1) [17.4, 29.6] 29.8 (21.6) [20.3, 39.1] 6.2 (15.7) [−0.5, 13.1]	20.6 (9.7) [16.6, 24.7] 36.4 (27.3) [24.8, 47.8] 15.7 (25.7) * [4.8, 26.6]	22.9 (13.9) [16.9, 29.1] 34.3 (25.1) [23.3, 45.1] 11.3 (26.8) * [−0.2, 22.8]	0.209
Cereals (g/day)	Baseline 6 months ▲	136.1 (65.5) [107.7, 164.4] 144.7 (56.1) [120.5, 168.9] 8.7 (88.3) [−29.5, 46.8]	108.5 (63.7) [81.5, 135.3] 113.6 (57.7) [89.2, 138.1] 5.1 (62.2) [−21.1, 31.4]	139.4 (58.7) [113.9, 164.7] 143.8 (56.7) [119.2, 168.3] 4.4 (74.9) [−27.9, 36.8]	0.937
Dairy (g/day)	Baseline 6 months ▲	324.4 (215.1) [231.4, 417.4] 286.3 (142.1) [224.8, 347.7] −38.1 (230.2) [−137.6, 61.4]	312.6 (182.4) [235.5, 389.5] 299.4 (151.5) [235.4, 363.4] −13.1 (164.9) [−82.7, 56.5]	288.6 (211.1) [197.3, 379.9] 346.5 (243.6) [241.1, 451.8] 57.9 (188.5) [−23.6, 139.3]	0.467
Meat (g/day)	Baseline 6 months ▲	191.8 (89.8) [152.9, 230.6] 123.1 (56.6) [98.5, 147.5] −68.8 (85.6) * ^d e^ [−105.7, −31.7]	166.5 (71.1) [136.5, 196.5] 143.8 (60.5) [118.2, 169.3] −22.7 (45.7) ^d^ [−42.1, −3.4]	151.7 (67.9) [122.3, 181.1] 136.2 (77.1) [102.8, 169.4] −15.6 (65.2) ^e^ [−43.8, 12.6]	0.008 ^
Olive oil (g/day)	Baseline 6 months ▲	39.6 (19.3) ^b^ [31.2, 47.9] 38.6 (15.5) [31.8, 45.3] −1.1 (18.8) [−9.1, 7.1]	32.9 (23.2) [23.1, 42.7] 32.2 (17.9) [24.5, 39.7] −0.8 (25.5) [−11.5, 9.9]	23.8 (12.1) ^b^ [18.6, 29.1] 30.2 (13.9) [24.1, 36.2] 6.3 (12.2) [1.1, 11.6]	0.596
Fish (g/day)	Baseline 6 months ▲	96.5 (78.4) [62.5, 130.3] 130.7 (70.9) [100–1, 161.3] 34.2 (58.8) * [8.8, 59.6]	95.5 (58.7) [70.6, 120.3] 140.9 (86.6) [104.3, 177.4] 45.4 (62.5) * ^f^ [18.9, 71.7]	112.6 (70.7) [82.1, 143.1] 114.2 (67.6) [84.9, 143.4] 1.6 (48.1) ^f^ [−19.1, 22.3]	0.050
Nuts (g/day)	Baseline 6 months ▲	8.8 (11.7) [3.7, 13.9] 19.2 (22.8) ^b^ [9.3, 29.1] 10.3 (22.3) [0.7, 19.9]	11.2 (14.9) [4.9, 17.5] 17.6 (15.3) ^c^ [11.1, 24.1] 6.4 (19.1) ^f^ [−1.7, 14.4]	17.9 (17.2) [10.4, 25-3] 41.4 (31.2) ^b c^ [27.9, 54.8] 23.5 (33.9) * ^f^ [8.8, 38.1]	0.049 ^
Sweets and pastries (g/day)	Baseline 6 months ▲	30.1 (44.5) [10.8, 49.3] 6.5 (7.8) [3.1, 9.8] −23.7 (44.9) * ^e^ [−43.1, −4.2]	17.9 (22.3) [8.4, 27.3] 11.7 (16.6) [4.7, 18.7] −6.2 (10.5) [−10.5, −1.7]	9.2 (9.4) [5.1, 13.2] 14.6 (28.9) [2.1, 27.1] 5.4 (27.8) ^e^ [−6.6, 17.4]	0.013 ^
Non-processed foods (g/day)	Baseline 6 months ▲	1245.1 (375.1) [1082.8, 1407.2] ^b^ 1334.9 (347.2) ^b^ [1194.7, 1495.1] 99.8 (337.7) [−46.1, 245.8]	1317.7 (457.9) [1124.3, 1368.8] 1487.8 (281.7) [1368.8, 1606.7] 170.1 (414.8) * [−5.1, 345.2]	1530.1 (619.2) [1262.3, 1797.9] ^b^ 1646.5 (605.7) ^b^ [1384.5, 1908.4] 116.4 (271.5) [−1.1, 233.7]	0.575
Low-processed foods (g/day)	Baseline 6 months ▲	63.9 (34.3) ^a b^ [49.1, 78.7] 44.1 (17.3) [36.5, 51.5] −19.8 (30.2) * ^d e^ [−32.8, −6.7]	40.4 (24.1) ^a^ [30.2, 50.5] 36.1 (18.4) [28.2, 43.8] −4.4 (25.1) ^d^ [−14.9, 6.2]	36.8 (15.1) ^b^ [30.3, 43.4] 42.1 (24.3) [31.5, 52.5] 5.2 (20.8) ^e^ [−3.7, 14.2]	0.008 ^
High-processed foods (g/day)	Baseline 6 months ▲	349.5 (203.2) [261.6, 437.3] 225.4 (96.4) [183.7, 267.1] −124.1 (179.7) * ^d^ [−201.8, −46.4]	291.3 (166.8) [220.8, 361.7] 261.6 (182.4) [184.5, 338.5] −29.7 (142.5) ^d f^ [−89.8, 30.4]	482.6 (425.1) [298.7, 666.3] 272.1 (170.7) [198.2, 345.9] −210.5 (373.9) * ^f^ [−372.1, −48.7]	0.043 ^
Ultra-processed food (g/day)	Baseline 6 months ▲	492.6 (325.4) ^a b^ [351.8, 633.2] 92.7 (93.3) [52.3, 133.1] −399.8 (302.9) * ^d e^ [−530.7, 268.8]	179.2 (156.4) ^a^ [113.1, 245.2] 115.3 (151.4) [51.3, 179.2] −63.9 (55.1) ^d f^ [−87.2, −40.6]	124.2 (112.7) ^b^ [75.4, 172.9] 165.4 (162.7) [95.1, 235.7] 41.1 (67.7) ^e f^ [11.8, 70.4]	<0.001 ^
Total energy (kcal/day)	Baseline 6 months ▲	2594.9 (634.1) ^a^ [2320.7, 2869.1] 1989.7 (416.2) ^b^ [1809.7, 2169.6] −605.3 (615.1) * ^d e^ [−871.2, 339.2]	2055.2 (499.2) ^a^ [1844.3, 2265.9] 1965.9 (391.9) ^c^ [1800.4, 2131.4] −89.2 (523.8) ^d^ [−310.4, 131.9]	2216.2 (568.5) [1970.3, 2462.1] 2425.8 (781.4) ^b c^ [2087.8, 2763.7] 209.5 (467.8) ^e^ [7.2, 411.8]	<0.001 ^

Abbreviations: UPF: ultra-processed food. SD: standard deviation. CI: confidence interval. Med-diet: Mediterranean diet (evaluated with the 17-item energy-reduced questionnaire). ^§^ Differences in %UPF consumption between baseline and at 6 months follow-up distributed across tertiles. Confidence intervals are calculated at the 95% confidence level and are represented by the values in brackets []. ▲ Change between baseline and 6 months. ^ Size effect was calculated using η^2^*p* (partial eta squared). * Time-related changes within the group. (a, b, c): letters denote group differences at each time point (ANOVA). (d, e, f): letters denote intergroup differences in time-related changes (post hoc analysis). GLM was adjusted by HOMA-IR and physical activity.

## Data Availability

There are restrictions on the availability of data for this trial, due to the signed consent agreements around data sharing, which only allow access to external researchers for studies following the project purposes. Requestors wishing to access the trial data used in this study can make a request to pep.tur@uib.es.

## Clinical Trials Registration

This trial was registered at ClinicalTrials.gov with the registry number NCT04442620 (https://clinicaltrials.gov/ct2/show/NCT04442620, accessed on 22 February 2022).
